# Speckle tracking echocardiography in primary mitral regurgitation: should we reconsider the time for intervention?

**DOI:** 10.1007/s10741-021-10100-1

**Published:** 2021-04-07

**Authors:** Maria Concetta Pastore, Giulia Elena Mandoli, Aleksander Dokollari, Gianluigi Bisleri, Flavio D’Ascenzi, Ciro Santoro, Marcelo Haertel Miglioranza, Marta Focardi, Luna Cavigli, Giuseppe Patti, Serafina Valente, Sergio Mondillo, Matteo Cameli

**Affiliations:** 1 Department of Medical Biotechnologies, Division of Cardiology, University of Siena, Viale Bracci 16, Siena, Italy; 2 Maggiore Della Carità Hospital, University of Eastern Piedmont, Novara, Italy; 3 Division of Cardiac Surgery, University of Toronto, Toronto ON, Canada; 4 Department of Advanced Biomedical Science, Federico II University Hospital Naples, Naples, Italy; 5 Cardiology Institute of Rio Grande Do Sul, Porto Alegre, Brazil

**Keywords:** Mitral regurgitation, Speckle tracking, Echocardiography, Surgery, Treatment, Timing

## Abstract

Thanks to the improvement in mitral regurgitation (MR) diagnostic and therapeutic management, with the introduction of minimally invasive techniques which have considerably reduced the individual surgical risk, the optimization of the timing for MR “open” or percutaneous surgical treatment has become a main concern which has highly raised scientific interest. In fact, the current indications for intervention in MR, especially in asymptomatic patients, rely on echocardiographic criteria with high severity cut-offs that are fulfilled only when not only mitral valve apparatus but also the cardiac chambers’ structure and function are severely impaired, which results in poor benefits for post-operative clinical outcome. This led to the need of new indices to redefine the optimal surgical timing in these patients. Speckle tracking echocardiography provides early markers of cardiac dysfunction due to subtle myocardial impairment; therefore, it could offer pivotal information in this setting. In fact, left ventricular and left atrial strains have already shown evidence about their usefulness in recognizing MR impact not only on symptoms and quality of life but also on cardiovascular events and new-onset atrial fibrillation in these patients. Moreover, right ventricular strain could be used to identify those patients with advanced cardiac damage and different grades of right ventricular dysfunction, which entails higher risks for cardiac surgery that could overweigh surgical benefits. This review aims to describe the importance of reconsidering the timing of intervention in MR and to analyze the potential additive value of speckle tracking echocardiography in this clinical setting.

## Introduction

Primary mitral regurgitation (MR) is a degenerative disease involving the mitral valve (MV) with progressive worsening of severity and poor long-term prognosis if untreated, in terms of lower survival, impaired quality of life, and increased burden of atrial fibrillation (AF) and heart failure (HF) symptoms. The only effective treatment for severe primary MR is MV repair or replacement. For asymptomatic patients with primary MR, current indications for surgical treatment rely on bidimensional echocardiographic criteria.

In fact, European and American guidelines [1, 2] recommend referring patients with severe primary MR to surgical treatment in presence of either symptoms or overt left ventricular (LV) dilatation and dysfunction, high pulmonary pressure, left atrial (LA) dilatation, and/or AF (Table 1), in order to spare the risks of cardiac surgery. Using these parameters, often early structural alterations due to high filling pressures are not recognized, since LV and LA are still capable to face hemodynamic overload; moreover, the cut-off values recommended for these parameters to refer patients to MV repair/replacement are too high, corresponding to severe structural and functional cardiac impairment. As a consequence, there is the risk to treat patients only in the last stages when cardiac chambers are irreversibly damaged. In fact, patients with chronic MR remain asymptomatic for long periods, due to adaptive remodeling of the LV and LA, even in the presence of severe degrees of regurgitation [3].

**Table 1:** Indications for mitral valve intervention in patients with chronic primary mitral regurgitation according to 2017 ESC guidelines and the newest ACC/AHA guidelines [1, 2]

*ESC 2017 guidelines*	*ACC/AHA 2020 guidelines*
**MV surgical treatment (repair, if possible)**	
Symptomatic patients with severe chronic MR and: – LVEF > 30% *– And/or* refractory to medical therapy with high likelihood of durable valve repair *– Or* with severe LV dysfunction and/or dilatation (LV EF < 30% and/or LVESD > 55 mm) with high likelihood of successful repair and low comorbidity	Symptomatic patients with severe chronic MR, corresponding to *Stage D*: *− either* moderate/severe LA enlargement *− or* LV enlargement *− or* pulmonary hypertension regardless of LVEF − if attributable to *rheumatic valve disease* at a Comprehensive Valve Center by an experienced team and if a durable and successful repair is likely
Asymptomatic patients with severe chronic MR and: − LV dysfunction (LVEF ≤ 60%) or severe dilatation (LVESD ≥ 45 mm) *− And/or* new onset AF or pulmonary hypertension (sPAP > 50 mmHg) *− And/or* high likelihood of durable repair *and* low surgical risk *and* LV dilatation (LVESD ≥ 40 mm) *and* flail leaflet *or* LA severe dilatation (LAVI ≥ 60 ml/m^2^) at sinus rhythm	Asymptomatic patients with severe (*either* moderate/severe LA enlargement *or* LV enlargement *or* pulmonary hypertension at rest or during exercise) with chronic MR and: − LV systolic dysfunction (LVEF ≤ 60%, LVESD ≥ 40 mm) (*Stage C2*) − normal LV systolic function (LVEF ≥ 60% and LVESD ≤ 40 mm) (*Stage C1*), when the likelihood of a successful and durable repair without residual MR > 95% with < 1% expected mortality when it can be performed at a Primary or Comprehensive Valve Center (IIa) or to consider with a progressive increase in LV size or decrease in EF on ≥ 3 serial imaging studies (IIb)
**MV percutaneous edge-to-edge repair**	
Severe chronic MR patients with: − Symptoms refractory to medical therapy and severe LV dysfunction (LVEF < 30%) [also considering LV assist devices, CRT, cardiac restraint devices, heart transplantation] − Symptoms who fulfill the echocardiographic criteria of eligibility with high- surgical risk *or* who are judged inoperable by the Heart Team	Severe chronic MR patients and: − severely symptomatic patients (NYHA class III or IV) with high or prohibitive surgical risk if mitral valve anatomy is favorable for the repair procedure and patient life expectancy is at least 1 year

*AF* atrial fibrillation, *CRT* cardiac resynchronization therapy, *LA* left atrium, *LAVI* left atrial volume index, *LV* left ventricular, *LVEF* left ventricular ejection fraction, *LVESD* left ventricular end-systolic diameter, *MR* mitral regurgitation, *MV* mitral valve, *sPAP* systolic pulmonary artery pressure

On the other hand, undertreated severe MR could lead to a chronic overload state which reflects first on the LA, then on pulmonary circulation and, consequently, on the right ventricle (RV). This chamber, being less muscular and strongly load- and LV function-dependent, is less capable to sustain hemodynamic overload for a long period; therefore, it is not uncommon that patients with severe MR present RV dysfunction. Studies have suggested how RV dysfunction, detected by noninvasive parameters as surrogate of pre-operative overall heart function, adds great value as a marker of poor post-operative outcome after MV surgery [4]; thus, it should not be overlooked in the clinical decision-making of these patients.

However, in the last years, the development of new minimally invasive surgery and percutaneous MV repair has considerably reduced surgical risks [5]. Currently, MV repair is the preferred treatment over MV replacement due to lower peri-operative mortality and post-operative risk of bleeding and endocarditis [6]; notably, an early surgery has shown to provide better long-term survival and lower HF rates [7]. Percutaneous MV repair is recommended only in the case of high surgical risks and therefore is mainly adopted as therapeutic approach in patients with functional MR. However, the continuously growing evidence on favorable outcomes with MitraClip in patients with degenerative MR would possibly lead to the spread of its use towards early MV repair in younger patients who would clearly benefit from avoiding oral anticoagulation in case of mechanical prosthesis and biological low-durable prosthesis, regardless of the surgical risk. These patients should be adequately selected, and the advances in cardiovascular imaging could allow a better characterization of MV disease and its consequent myocardial alterations, in order to obtain a tailored therapeutic decision-making.

The aim of this review is to discuss the reasons for the potential need of introducing new echocardiographic indices to redefine the timing for MR surgery in light of the new evidence.

## Need for new criteria for mitral surgery

The issue of MR surgical timing has been widely debated in the last decades [8].

A previous study by Ling et al. has clearly shown how late surgery of primary MR is characterized by lower survival compared to patients undergoing the operation in presence of preserved LV ejection fraction (EF) and lower grades of symptoms [9]. It is arguable that when LV EF reduction and LA dilatation are detected in patients with primary MR, myocyte fibrotic process has already established in the cardiac chambers, and often it has also been reflected on pulmonary circulation and also right heart [10]. Therefore, in these phases, the initial hemodynamic pathologic condition caused by valvular heart disease has turned into final structural heart disease due to left chambers remodeling, with permanent consequences such as the predisposition to the development of HF and AF [3].

To date, none of the parameters used for surgical indications have a defined trend towards progression in the clinical history of patients with MR. In fact, some patients remain asymptomatic and stable for years, whereas others suddenly develop symptoms with or without the presence of echocardiographic criteria. For example, a recent study by Ma et al. showed that patients followed for around 4.5 years for MV prolapse having mild-to-moderate MR and normal EF. Among them, none of the patients with mild MR progressed to severe MR, whereas 50% with moderate MR progressed to severe MR, but no clinical variables or echocardiographic parameters predicted the progression of the disease apart from the dilatation of mitral annulus (sensitivity 100%, and specificity 63.8%) [11].

The absence of clear predictors of MR progression and clinical deterioration makes clinicians rely in favor of possibly misleading serial clinical and echocardiographic monitoring while on medical therapy, rather than expose the patient to surgical risks.

However, in the last years, the advances in cardiac surgery and interventional cardiology have shown how the peri-operative risk can be reduced by the application of mini-invasive surgical techniques [6] and percutaneous MV repair [5]. The latest has been showing evolving results in terms of safety and efficacy of multiple novel devices (i.e., Mitraclip, Tendyne, Pascal), even if interventional approaches are still preferred in high-risk patients with specific favorable anatomical characteristics of the MV [12]. These patients are generally selected by a multidisciplinary heart team, including general and interventional cardiologists, cardiac surgeons, cardiac imagers, HF specialists, and cardiac anesthesiologists.

Mitraclip is currently the most studied, as it has shown satisfactory results for the treatment of primary and secondary MR [13, 14]. The EVEREST II trial, performed in patients with primary MR randomized to Mitraclip or surgical (86% minimally invasive) treatment, showed that 12-month mortality and rates of ≥ 3 grade residual MR were similar in the two groups (6% and 20%, respectively), with higher complications (e.g., blood transfusions) in the surgical arm and higher rate of subsequent surgery for MV dysfunction in the MitraClip arm (20% vs. 2%). This was confirmed in the subsequent 5-year follow-up, where patients who had received MitraClip still have higher rates of repeat surgery and residual MR vs those in the surgical arm, but with comparable overall mortality. However, the longer-term follow-up of the EVEREST II trial demonstrated significant benefits in terms of quality of life and a decrease from 45 to 5.7% of NYHA class III/IV symptoms at 4 years after MitraClip implantation in patients at prohibitive risk of MV surgery [15]. Importantly, a timely MV intervention has also demonstrated improvement in LV and LA reverse remodeling after successful MR reduction by MitraClip intervention [16].

These data support the safety and effectiveness of MitraClip in primary MR, highlighting the importance of appropriate patient’s selection in order to improve peri-procedural results and reduce the subsequent rates of re-intervention. However, an early surgery, performed in patients with intermediate stages of chronic MR and without irreversible damage of MV anatomy and left cardiac chambers would probably lead to better results in terms of durability.

Suri et al., with the development of a Mitral Regurgitation International Database, including more than one thousand patients with flail-related MV regurgitation and without class I criteria for surgery, have found that an early surgery (< 3 months from diagnosis) vs. medical treatment strongly influenced long-term survival (86% vs. 69% 10-year survival, hazard ratio 0.66), with a 53% reduction in 5-year mortality (*p* < 0.001) and a 71% decrease of HF risk (7% vs. 23% at 10 years, *p* < 0.001) [7].

For these reasons, many clinicians are wondering if updating the commonly used criteria to refer patients to MV surgery by introducing newer indices of early MR-related left heart impairment would allow us to reconsider the timing for MV surgery and provide a timely treatment. This would probably lead to post-operative benefits, in terms of functional recovery and clinical outcome.

## Advanced echocardiography

The spread of advanced echocardiography has been an important step for a more accurate evaluation of valvular heart disease. In fact, speckle tracking, transesophageal, and three-dimensional echocardiography have become essential tools in the hands of cardiac imagers for diagnosis, surgical indication, planning of intra-operative assistance, and follow-up assessments in patients with valve heart disease [17]. Two-dimensional (2D) speckle tracking echocardiography (STE) is one of the mostly available and easy-to-use advanced echocardiographic techniques, being performed through a semi-automatic rapid analysis of basically acquired 2D echocardiographic images [18]. It has shown to be an early diagnostic and prognostic marker in several clinical scenarios [19] and has been used by several authors for the evaluation of patients with MR, since it is able to provide an early assessment of subtle myocardial fibers impairment [20].

## Left ventricular global longitudinal strain in mitral regurgitation 

Several authors showed the important role of LV global longitudinal strain (GLS) over LV EF as an index of subclinical cardiac dysfunction in MR. Its use might lead to an improvement in the detection of LV damage in these patients as well as a more accurate prognostic evaluation for surgical referral.

Kislitsina et al. demonstrated that preoperative strain measurements in 119 patients with degenerative MR undergoing surgical treatment represented the most accurate parameter in detecting an early LV dysfunction after MV surgery, despite preserved LV EF [21].

LV strain was able to identify subclinical LV dysfunction in asymptomatic patients with primary MR undergoing surgical (*n* = 30/71 patients) or medical treatment and preserved LV EF undergoing exercise echocardiography, and to predict post-operative LV dysfunction [22, 23]. Furthermore, in a cohort of patients with moderate to severe organic MR undergoing MV repair, a LV GLS > −19.9% was the strongest, independent predictor of long-term post-operative LV dysfunction (LV EF < 50%) (odds ratio 23.16, *p* < 0.001) [24].

In a large cohort (*n* = 506) of patients with severe chronic MR, Kim et al. showed that LV GLS was a significant predictor of cardiac events (hospitalization for HF, re-intervention for MV surgery failure, cardiac death) also with multivariate analysis, regardless of the presence of LV dysfunction, AF, and the type of surgery. Moreover, reduced GLS was also associated with all-cause mortality in this study (HR 1.068, 95% CI 1.003 to 1.136; *p* = 0.040) [25]*.*

Another big study by Hiemstra et al. [26] conducted in 593 patients undergoing surgical intervention for severe MR showed that preoperative LV GLS ≥ - 20.9% was associated with worse survival over long-term follow up (6.4 [3.6–10.4 years]), providing a considerable incremental value over a clinical model including age, AF, NYHA class ≥ II, estimated glomerular filtration rate, LV end-diastolic diameter, LVEF, systolic pulmonary artery pressure) for the prediction of all-cause mortality (C-statistic 0.74 to 0.77).

Interestingly, Singh et al. proved that, among patients referred for surgical treatment of severe MR, those with pre-operative preserved GLS had higher chances of postoperative LVEF > 50%, regardless of baseline LVEF also in MR of rheumatic etiology [27]. Also, Alashi et al. found that the combination of LV GLS and brain natriuretic peptide (BNP) was associated with persistent postoperative LV dysfunction and increased mortality, independent of other clinical or echocardiographic parameters in asymptomatic patients with significant primary mitral regurgitation and preserved systolic function undergoing mitral valve surgery [28].

To conclude, a recent meta-analysis involving 2358 patients (from 8 studies) with severe MR and preserved LVEF highlighted that a reduced LV GLS before surgery was a predictor of worse postoperative survival (HR = 1.13, 95%CI: 1.02–1.26) and LVEF [29].

Table 2 provides an overview on the main evidence on the prognostic role of LV strain in MR currently available in literature. These results all suggest that GLS might have an additional value over conventional measures to optimize the timing of surgery as it is affected much earlier than LVEF.

**Table 2:** Current evidence supporting the application of left ventricular global longitudinal strain for the prognostic evaluation of primary mitral regurgitation

Reference	Study cohort	Outcome	Strain parameter	Accuracy
Kislitsina et al. Ann Thorac Surg. 2020 [21]	520 pts (119 with STE) undergoing MV surgery for severe degenerative MR with LVEF ≥ 60%	Early postoperative LV dysfunction and medium-term overall survival	LV strain	HR = 2.314 (1.528, 3.504), *p* < 0.001
Cho et al. Korean Circ J. 2016 [23]	43 pts with chronic severe MR and preserved LVEF scheduled for mitral valve replacement or repair (51.7 ± 14.3 years)	Postoperative LV remodeling (reduction in LVEF or increase of LVEDD at 3 months)	LV GLS > −20.5%	Sensitivity 0.70, specificity 0.75 OR = 2.440 (1.259–4.729), *p* = 0.008
Witowski et al. Eur Heart J Cardiovasc Imaging 2013 [24]	233 pts with moderate–severe organic MR who underwent successful MV repair (61 ± 12 years)	Postoperative LV dysfunction (LVEF < 50%) at long-term follow up (34 ± 20 months)	LV GLS > −19.9%	Sensitivity = 90%, specificity = 79% OR = 23.16 (95% CI: 6.53–82.10, *p* < 0.001) at multivariate analyis
Kim et al. JACC Cardiovasc Imaging. 2018 [25]	506 pts with severe primary MR who underwent MV surgery (58.5 ± 13.7 years)	Cardiac events: admission for worsening HF, reoperation for failure of MV surgery, cardiac death	LV GLS > −18.1%	HR: 1.229 (*p* < 0.001) regardless of the presence of LV dysfunction, AF, type of surgery
Hiemstra et al. JACC Cardiovasc Imaging 2020 [26]	593 pts with severe primary MR who underwent MV surgery (65 ± 12 years)	Primary endpoint: all-cause mortality Secondary endpoint: cardiovascular death, HF hospitalizations, cerebrovascular accidents	LV GLS > −20.6%	HR: 1.13 (95% CI: 1.06 to 1.21; *p* < 0.001) for all-cause mortality
Alashi et al. Circ Cardiovasc Imaging 2016 [28]	448 pts asymptomatic patients with MR ≥ 3+ and preserved LVEF (61 ± 12 years)	All-cause mortality Post-operative LV dysfunction (LVEF < 50%)	LV GLS	HR = 1.17 (1.08–1.27); *p* < 0.001 for every unit increase *χ*^2^ increase from 31 to 47 to 61, (*p* < 0.01) For addition of lnBNP and LV-GLS to a standard clinical model (STS score and RVSP) OR = 1.22 (1.11–1.34) *p* < 0.001 for every unit increase
Mentias et al. J Am Coll Cardiol 2016 [30]	737 asymptomatic pts with MR ≥ 3+ (58 ± 13 years)	All-cause mortality	LV GLS > −21.7%	-HR: 1.60; 95% CI 1.47 to 1.73 -C-statistic increase from 0.69 to 0.78 (0.65 to 0.90; *p* < 0.01) adding GLS to a clinical model (STS score, indexed LV-ESD, RVSP, and mitral ERO) -Long-term survival 1.4% vs. 16% (*p* < 0.001) with Kaplan Meier analysis
Pandis et al. J Am Soc Echocardiography 2014 [32]	130 pts undergoing MV repair for severe MR (57 ± 14 years)	Changes in LVEF 6 months aftery surgery - > postoperative LVEF reduction > 10% - > postoperative LVEF reduction > 10% with LVEF < 50%	LV GLS LV GCS LV GRS	*r* = −0.71, *p* < 0.0001 - > OR = 0.80; *p* < .001 - > AUC = 0.93; *p* < .001 *r* = −0.22, *p* = 0.01 No correlation
Mascle et al. J Am Soc Echocardiography 2012 [33]	88 pts with severe MR 63 6 13 years	Postoperative LV dysfunction (LVEF < 50% after 6 ± 1 months)	LV GLS > −18%	AUC 0.70 (SE 53%, SP 79%) OR, 4.2; 95% CI, 1.4–13; *p* = .009 at multivariate analysis
Fukui et al. J Am Soc Echocardiography 2020 [31]	155 pts undergoing transcatheter edge-to-edge mitral valve clip implantation (mean age, 83 ± 7 years; 137 with degenerative MR and 18 with functional MR)	All-cause mortality	GLS > −14.5%	AUC = 0.60 (95% CI 0.49–0.71) *χ*^2^ = 6.36, *p* = 0.012 Independent predictor at multivariate analysis in 4 models, regardless of STS-PROM score, LVEF, functional MR, estimated glomerular filtration rate, frailty

*AUC* area under curve, *ERO* effective regurgitant orifice area, *ESD* end-systolic diameter, *HF* heart failure, *LV* left ventricular, *LVEF* left ventricular ejection fraction, *MR* mitral regurgitation, *MV* mitral valve, *RVSP* right ventricular systolic pressure, *SE* sensitivity, *SP* specificity, *STE* speckle tracking echocardiography, *STS* Society of Thoracic Surgeons

Even though its use could be still limited by the lack of disease-specific cut-off values and high dependence on acoustic window, LV GLS is a useful parameter to be included when considering early surgery in patients with severe mitral regurgitation and normal LVEF, providing important information on postoperative outcome.

**Fig. 1:**
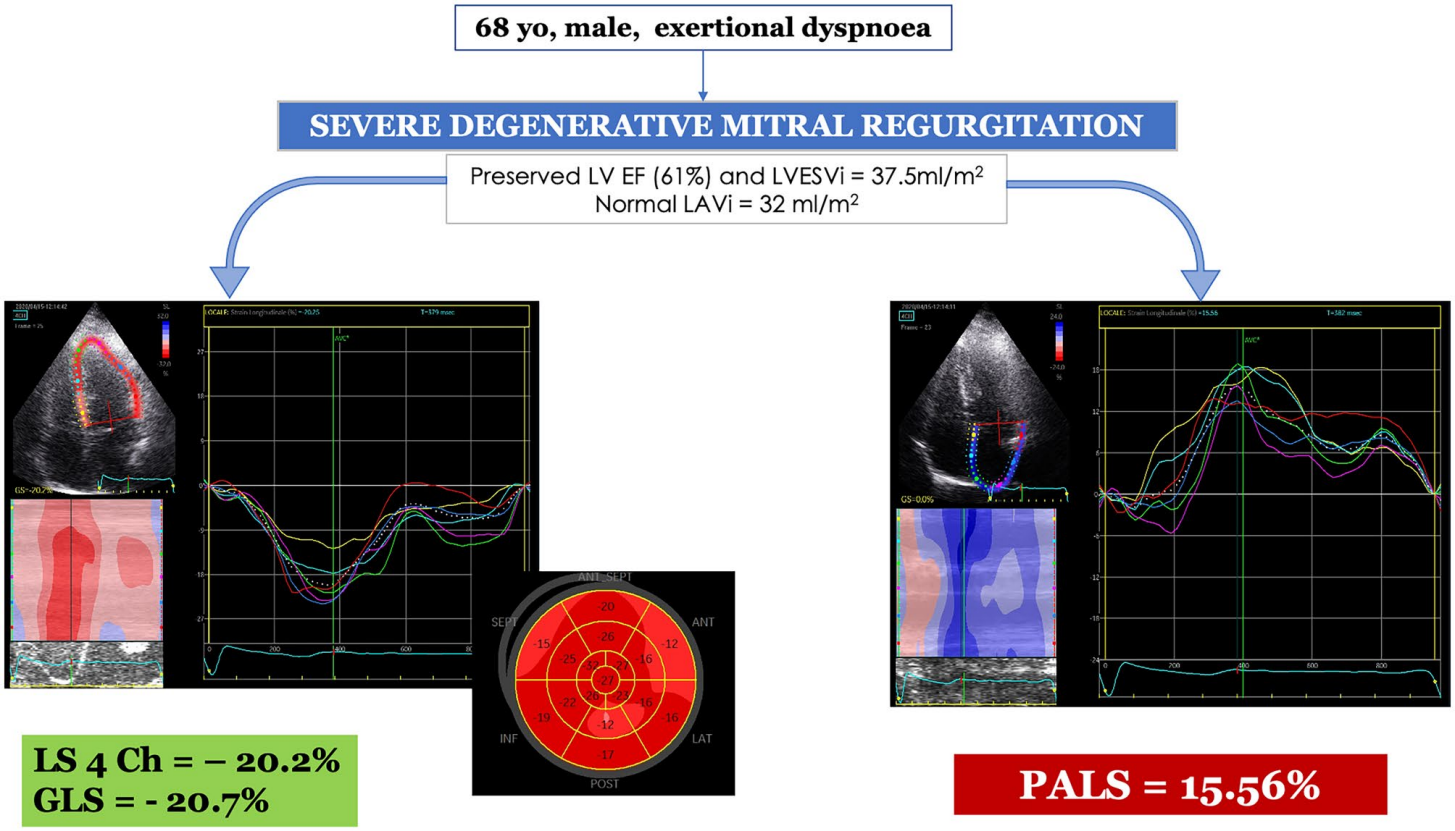
Representative case of a patient with severe mitral regurgitation symptomatic for exertional dyspnea, referred for cardiac surgery. Basic echocardiography showed preserved left ventricular (LV) dimensions and function (LV ejection fraction = 61%, LV end-systolic volume index 35 ml/m^2^) and normal left atrial (LA) volume (LA volume index = 32 ml/m^2^). Interestingly, speckle tracking echocardiography detected left atrial dysfunction (considerably reduced peak atrial longitudinal strain = 15%, *right*) with preserved LV strain (4-chamber longitudinal strain = −20.2%, global longitudinal strain = −20.7%, *left*). GLS global longitudinal strain, LS longitudinal strain, LVEF left ventricular ejection fraction, LVESVi left ventricular end-systolic volume index, PALS peak atrial longitudinal strain

However, often LA strain is affected previously than LV strain (Fig. 1) in patients with severe MR who have not developed LV dysfunction yet. Accordingly, it was described as superior to LV GLS in several studies evaluating MR severity and prognosis [31, 34–36], probably because LA is the direct target chamber affected by the chronic volume overload deriving from worsening degenerative MR [3]; therefore, its involvement occurs at an earlier stage compared to the LV.

## Left atrial strain as the earliest prognostic marker

LA strain is actually considered a hallmark of LV filling pressures and diastolic dysfunction [37], related to the amount of invasively assessed myocardial fibrosis [35, 38, 39]. LA strain follows LA deformation all over the cardiac cycle, allowing to quantify LA reservoir, conduction, and contractile properties. Peak LA strain (PALS), a measure of LA reservoir function calculated using QRS as reference [40], is the most widely used parameter, having a more robust evidence compared to LA conduit and contractile strain, and being independent from the presence of sinus rhythm (i.e., conversely contractile strain is absent in AF due to the lack of LA contraction) [41].

**Fig. 2:**
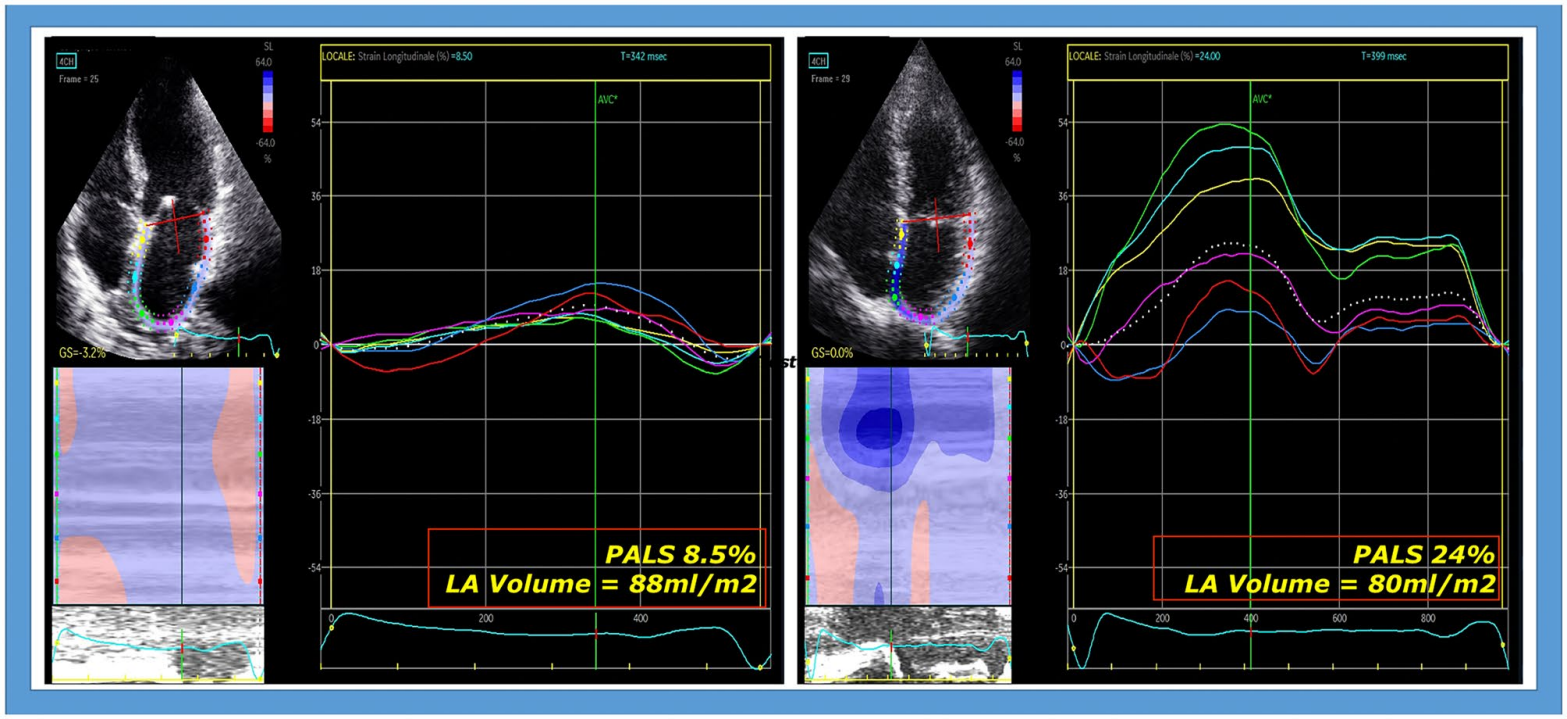
Representative case of early modification of left atrial (LA) deformation in a patient undergoing cardiac surgery for severe mitral regurgitation, showing pre- (left) and post- surgical (right) normalization of LA strain. In particular, LA strain improved from 8.5% to 24% in only 5 days after removing the hemodynamic stress on LA due to severe MR. LA volume did not significantly change early after surgery (pre-surgical LAVI 88 ml, post-surgical 80 ml)

PALS evaluation had a particular usefulness during pre-surgical evaluation of primary MR for the prediction of postoperative LA reverse remodeling and clinical outcome; in fact, PALS was independently associated to post-operative survival and functional capacity as assessed by NYHA class [42] and Borg scale [43] not only in patients with severe MR but also in those with lesser severity of the valvular disease [34, 44]. It could also be used to monitor post-operative cardiac changes, representing an early index of LA remodeling, which often occurs prior to LV volume reduction as a beneficial effect of the surgery (Fig. 2, representative case).

**Fig. 3:**
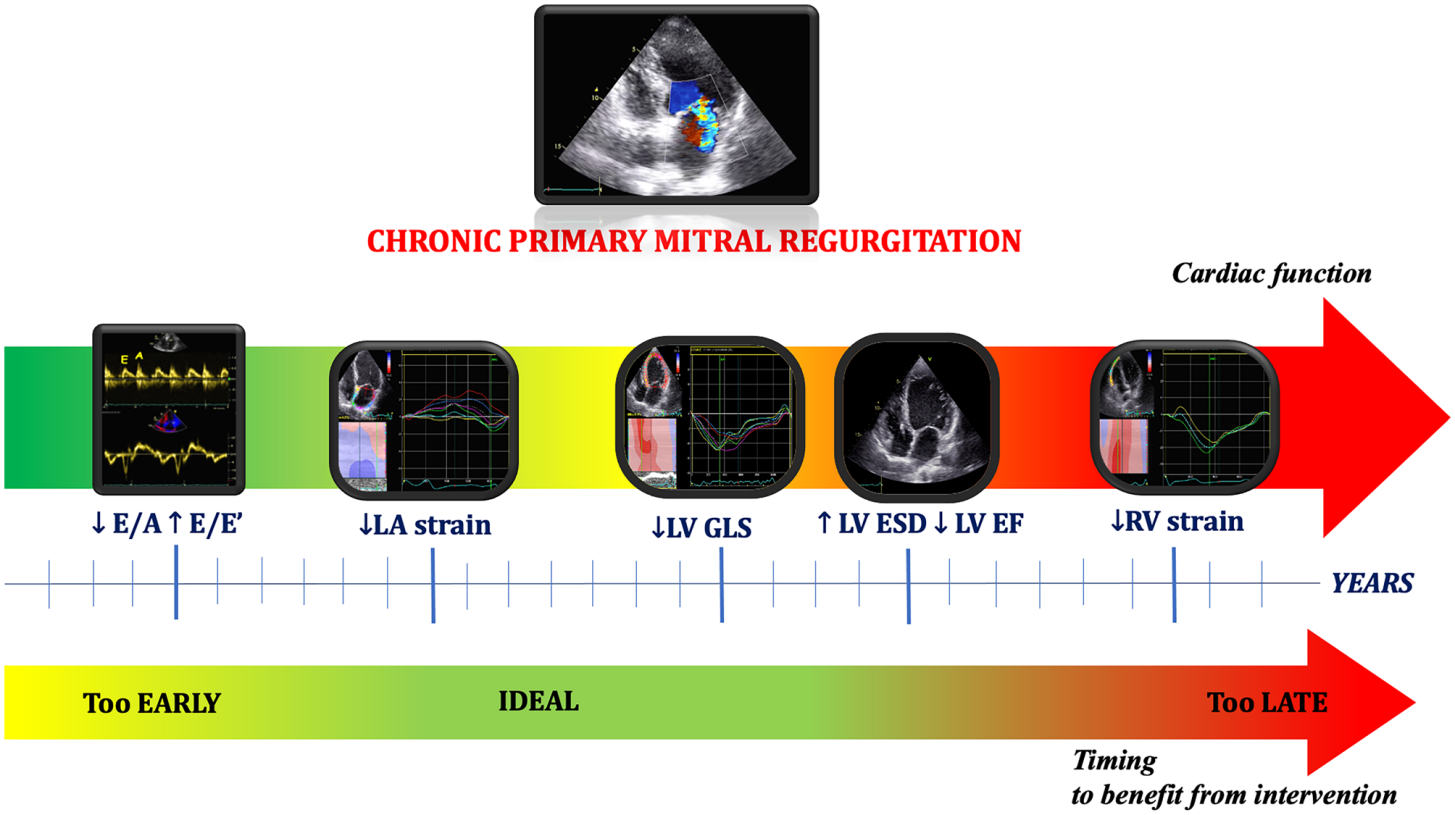
Echocardiographic indices changing over time in primary mitral regurgitation. Graphic timeline representing the progressive variation of echocardiographic parameters in the clinical history of mitral regurgitation, with corresponding ideal surgical timing in order to optimize the benefits related to mitral valve intervention. From the left, the initial raise in left ventricular (LV) filling pressures could be detected by a reduction of transmitral peak early diastolic velocity (*E*)/peak late diastolic velocity (*A*) ratio by pulsed-wave Doppler and a raise in E/average mitral annular velocity (E’) by tissue Doppler imaging. Later, initial left atrial (LA) damage and remodeling due to the chronic volume overload is detectable by a reduction of LA strain before its severe dilatation; this corresponds to the ideal timing for intervention in order to optimize clinical outcome and reduce the occurrence of arrhythmias. Then, after years of severe chronic mitral regurgitation, LV involvement takes place: initially, it consists in subtle myofibril damage, which is only detectable by LV longitudinal strain reduction. Later, LV overload leads to LV dilatation and dysfunction; hence, LV ejection fraction pathologically decreases; although this is the index mostly used for surgical referral, this identifies a consolidated left heart impairment, with low chances of reversal. Finally, when biventricular dysfunction occurs, the risks deriving from cardiac surgery (i.e. “late surgery”) overweigh possible benefits; for this reason, right ventricular strain could help identifying subtle right ventricular dysfunction in order to optimize surgical decision-making or choosing less-invasive therapeutic strategies

Notably, LA strain has also shown a reliable index of LA remodeling following MitraClip procedure over conventional LV and LA echocardiographic indices [45].

Moreover, PALS was the only resting echocardiographic parameter associated with exercise-induced pulmonary hypertension in a cohort of patients with asymptomatic MR [46]. PALS during exercise was related to survival and HF hospitalization in a cohort of 196 patients with primary or secondary MR [47].

Interestingly, some authors focused on the potential relationship of LA strain with indications for MV surgery: Debonnaire et al. found a correlation between LA strain and current surgical criteria: symptoms, AF, and development of PH [48]; in patients with MV prolapse and with moderate-to-severe MR, Ring et al. showed that PALS and LA contractile strain were associated with time to mitral surgery (HR 3.06) and surgery-free survival (3-year survival 67 ± 6% for PALS > 28.5% vs 15 ± 6% for PALS < 28.5%), in patients MV prolapse and with moderate-to-severe MR [49].

Despite the occurrence of AF is considered a surgical criterion for MR, it is known that the development of AF is directly related to LA fibrosis [50]; therefore, it should be prevented as it means the presence of irreversible myocardial structural abnormalities. LA strain has been described as a marker for the development of paroxysmal and permanent AF, since myocardial fibrosis strongly affects LA deformation properties quantified by STE [51]. Moreover, LA strain proved to be associated with AF occurrence and to be progressively more impaired in patients with MR and repeated episodes of paroxysmal AF [52] and was also associated with the development of post-operative AF in patients undergoing MV surgery [53]. Therefore, LA strain could be useful to identify patients prone to develop AF in order to optimize risk stratification of patients with MR.

All these findings suggest the potential value of considering LA strain as an additive criterion for the surgical referral of patients with primary MR. Table 3 summarizes current evidence on the application of LA strain in patients with MR, also including proposed reference cutoff values before surgery.

**Table 3:** Current evidence supporting the application of left atrial strain for the evaluation of primary mitral regurgitation

Reference	Study cohort	Endpoint	Strain parameter	Accuracy
Candan et al. Int J Cardiovasc Imaging 2014 [54]	53 pts undergoing MV surgery for severe MR and preserved LVEF (45.7 ± 13.5 years)	LA reverse remodeling (LAVI decrease > 15%)	PALS	Beta = 0.54, *p* = .001 in multivariate linear regression analysis
Yang et al. J Med Ultrasound 2017 [55]	55 pts with severe chronic Carpentier II MR (57.2 ± 15.7 years)	LA remodeling (LAVI increase)	Δ: PALS rate	OR: 0.037, 95% CI 0.003–0.496
Cameli et al. Int J Cardiovasc Imaging 2019 [44]	276 pts with moderate asymptomatic primary MR (66 ± 8 years)	Cardiovascular events (AF, stroke/TIA, acute HF, cardiovascular death) over 3.5 ± 1.6 years	PALS < 35%	AUC = 0.87 40-month event-free survival: 90% for PALS > 35%; 78% for PALS 25–35%; 62% for PALS 15–25%; 9% for PALS < 15%
Kamijima et al. [46]	91 asymptomatic pts with degenerative MR (59.1 ± 13.1 years)	Exercise-induced pulmonary hypertension (PH) detected by exercise stress-echo Symptoms over 44 ± 21 months (HF hospitalization, AF, resting PH, MV surgery)	PALS < 26.9%	AUC 0.85% 3-year symptom-free survival: 79% for PALS > 26.9%; 45% for PALS < 26.9%
Yang et al. Int J Cardiol 2013 [42]	136 pts with chronic severe primary MR and preserved LVEF	HF symptoms assessed by NYHA class > II	PALS	OR 0.891, 95% CI 0.796–0.997
Mandoli et al. Int J Cardiol 2020 [43]	65 pts with severe primary MR undergoing MV surgery (71 ± 8 years)	-Primary endpoint: All-cause/cardiovascular death, HF hospitalizations -Secondary endpoint: postoperative functional capacity assessed by NYHA class / Borg CR10 scale -Association with LA fibrosis detected by atrial biopsy	PALS < 21%	AUC 0.78 5-year event-free survival: 90 ± 5% for PALS ≥ 21% vs 30 ± 9% for PALS < 21% *r*^2^ = 0.11 with NYHA and *r*^2^ = 0.1 with Borg CR10 *r*^2^ = 0.8 with LA fibrosis
Candan et al. Echocardiography 2013 [53]	53 pts undergoing MV surgery	Post-operative AF development	PALS	OR: 0.72 in multivariate analysis (95% CI 0.54–0.95)
Cameli et al. Int J Cardiovasc Imaging [35]	197 pts with degenerative MR (65.9 ± 14.8 years)	Number of paroxysmal AF episodes	PALS	*r* = 0.76; *p* < 0.0001 Beta = −0.591: *p* < 0.0001 in multivariate analysis

*AF* atrial fibrillation, *AUC* area under curve, *HF* heart failure, *PALS* peak left atrial strain, *LAVI* left atrial volume index, *LVEF* left ventricular ejection fraction, *MR* mitral regurgitation, *MV* mitral valve, *NYHA* New-York Heart association, *PH* pulmonary hypertension, *OR* odds ratio, *TIA* transient ischemic attack

Logically, these promising results require larger studies to be generalized. Moreover, some limitations of LA strain by STE should be kept in mind. First, it strongly depends on loading conditions and image quality. Furthermore, the lack of disease-specific cut-off values for reference and the fact that a universally method of execution has not been reached yet should be considered. However, several documents have been published in order to standardize its application among different vendors and countries [41, 56].

## Right ventricular dysfunction: too late for surgery?

In patients with severe MR, the gradual increase in left heart filling pressure due to progressive LV and LA structural and functional damage leads to pulmonary circulation overload and development of pulmonary hypertension [1]. The elevation of pulmonary pressure and RV afterload induce RV remodeling resulting in RV hypertrophy and/or RV dilatation. This process is faster than LV remodeling, since RV has lower thickness and its dependence from LV function and loading conditions make it easier to occur. These changes may lead to secondary tricuspid valve regurgitation and retrograde flow into the right atrium with further RV volume overload and RV function deterioration [10].

This pathophysiologic picture describes patients with advanced stages of MV disease and coexisting biventricular failure. This was confirmed by Bakkestrom et al., who found a significant inverse correlation between resting mean pulmonary artery pressure (detected by right heart catheterization) and both LV EF (*r* = −0.52; *p* = 0.02) and RV EF (*r* = −0.67; *p* < 0.01) in a subgroup of symptomatic subjects with degenerative MR, identifying a strict correspondence between the onset of symptoms and a state of pulmonary hypertension and biventricular dysfunction [57].

Moreover, some authors described a significant correlation between MR severity and RV dysfunction. Le Tourneau et al. demonstrated that RV EF was inversely correlated to mitral effective regurgitant orifice (*r* = −0.28, *p* = 0.012) and to regurgitant volume (*r* = −0.25, *p* = 0.021) [4]. Sabe et al. showed that MR severity was an independent predictor of RV EF in patients with ischemic MR. Interestingly, in the same investigation, for each 10% decrease in RV EF, there was a 17% increased risk of mortality (*p* < 0.001) [58].

RV structure and function have an important prognostic value in several clinical settings, especially in chronic HF, representing the marker of transition to advanced HF and worse outcome. Furthermore, most patients undergoing cardiac surgery present a variable degree of reversible or irreversible reduction in RV function, which in turn was associated with lower post-operative survival [59]. Therefore, having an already impaired RV function at pre-operative assessment should in some way discourage aggressive therapies due to the higher risk of mortality for worsening RV failure.

Accordingly, an accurate assessment of RV function during pre-operative routine evaluation of MR is essential: Chrustowicz et al. analyzed a young cohort of patients with organic, severe MR by basic echocardiography and measured tricuspid annular plane systolic excursion (TAPSE), and the peak systolic velocity of the lateral tricuspid annulus (S'); a pre-operative reduction of these indices predicted a significant post-operative LV dysfunction (10% decrease in LV EF) [60]. The same authors in a previous investigation demonstrated that pre-operative RV dilatation was an independent predictor of mortality and need for heart transplantation after MV repair [61].

Finally, Mordi et al. have shown how a comprehensive preoperative echocardiography assessment of LV diastolic and RV systolic function carries an independent prognostic value (HR 8.76 and HR 4.97 respectively, *p* < 0.01) in predicting 30-day postoperative mortality among patients undergoing redo valve surgery, increasing the prognostic power of EuroSCORE II at c-statistic from 0.76 to 0.83 [62].

Increasing evidence has evaluated the role of STE for assessing RV function. In fact, the use of free-wall RV longitudinal strain allows to accurately measure RV global function in a reproducible and less load- and translational-dependent way. It has been extensively applied in HF [63]; in particular, it has become a cornerstone for the selection of patient candidates to LV assisting device, as a reduced pre-implantation RV free-wall strain identifies patients with increased risk of developing RV failure in the early or late postoperative period [64]. Thus, RV STE may also represent a rapid and useful non-invasive tool for the evaluation of surgical referral of patients with severe MR, as revealing initial or established RV damage, it could improve the pre-operative risk stratification. However, timely evidence is needed on this topic.

Hence, a complete RV echocardiographic pre-operative evaluation should not be forgotten in patients with severe MR, comprehensive of basic echocardiographic dimensional and functional RV parameters (e.g., RV diameters, TAPSE, RV fractional area change) and, if possible, integrating advanced echocardiographic parameters, such as STE. Unfortunately, RV strain measurement could sometimes be challenging, due to the need of RV dedicated 4 chambers views, and poor acoustic windows with incomplete visualization of RV free wall.

Overall, a preserved vs compromised RV function should represent an important element to consider in the assessment of patients’ eligibility for surgical treatment of MR.

## The novel role of 3D echocardiography

In the last years, different software implementations have been developed to assess three-dimensional strain. Area strain is a new 3D speckle tracking parameter, calculated as the percentage decrease in the size of endocardial (or mid-myocardial) surface area, and determined by vectors of longitudinal and circumferential deformation at end-systole from its original area at end-diastole [64]. It represents a novelty in cardiac imaging; however, some authors have already focused on its potential usefulness in different clinical settings, including primary and secondary MR.

Casas-Rojo et al. studied 45 asymptomatic patients with secondary MR and LVEF > 60% undergoing 3D echocardiography, showing that they had lower values of global 3D strain compared to healthy subjects and that 3D area strain greater than −41.6% reached a hazard ratio of 4.41 (*p* = 0.004) for the prediction of cardiac events (dyspnea, LV EF < 60%, or admissions for heart failure) over a mean follow up of 23.2 ± 14.5 months [65]

Interestingly, Scandura et al. used 3D strain to detect myocardial changes after MitraClip implantation. In fact, despite the absence of changes in LVEF and LV stroke volume, they showed a significant improvement in LV deformation, with the real-time 3D global longitudinal strain value changing from −9.8 ± 4.1% at baseline to −11.0 ± 4.4% at follow-up (*p* = 0.018) [66].

Vitarelli et al. [67] 2D and 3D strain were used to analyze 33 undergoing MitraClip with moderate to severe or severe secondary mitral regurgitation. They detected significant improvements not only in LV 2D global longitudinal strain, 3D GLS, and 3D area strain, but also in 3D RV EF and 3D RV free wall longitudinal strain (all *p* < 0.05). Importantly, patients with pre-existing RV dysfunction showed lower increase in LV strain after clip implantation, confirming the importance of assessing RV performance also before percutaneous MR intervention. In fact, LV and RV 3D strain showed high discriminative values (AUC 0.87–0.91) in predicting lower benefits after the procedure with persistence of symptoms (NYHA > II) [68].

Despite these promising results, 3D strain value in clinical practice is still debatable not only due to the scarce evidence but also due to vendor-dependency and the lack of standardization. Therefore, its role in selecting patients for early surgery has not been fully established so far.

## Conclusions

The progresses in diagnostic and therapeutic management of MR have raised scientific controversies about the need of reconsidering surgical timing for these patients in order to improve their clinical outcome. The currently used criteria for referral to traditional MV operation or MV percutaneous intervention has shown some gaps in identifying patients who are developing subtle structural and functional LV and LA impairment, with sometimes consequent referral to MV repair/replacement when an irreversible myocardial damage has already occurred.

The integration of STE in clinical practice, particularly using LA strain as additive criteria to indicate or discourage surgery, may represent a useful tool to improve the clinical decision-making process related to the optimal timing of intervention in patients with MR.

Finally, the identification of RV dysfunction in patients with MR should be also considered an important step of pre-operative risk stratification in order to prevent a poorer outcome in the case of late surgery (Fig. 3).

## References

[1] Baumgartner H, Falk V, Bax JJ, De Bonis M, Hamm C, Holm PJ (2017). ESC/EACTS Guidelines for the management of valvular heart disease. Eur Heart J.

[2] Otto CM, Nishimura RA, Bonow RO, Carabello BA, Erwin JP 3rd, Gentile F, Jneid H, Krieger EV, Mack M, McLeod C, O'Gara PT, Rigolin VH, Sundt TM 3rd, Thompson A, Toly C (2021) 2020 ACC/AHA guideline for the management of patients with valvular heart disease: a report of the American College of Cardiology/American Heart Association Joint Committee on Clinical Practice Guidelines. Circulation. 143(5):e72-e227. Erratum in: Circulation. 2021;143(5):e229 10.1161/CIR.0000000000000923 33332150

[3] Cameli M, Incampo E, Mondillo S (2017). Left atrial deformation: useful index for early detection of cardiac damage in chronic mitral regurgitation. Int J Cardiol Heart Vasc.

[4] Le Tourneau T, Deswarte G, Lamblin N, Foucher-Hossein C, Fayad G, Richardson M, Polge AS, Vannesson C, Topilsky Y, Juthier F, Trochu JN, Enriquez-Sarano M, Bauters C (2013). Right ventricular systolic function in organic mitral regurgitation: impact of biventricular impairment. Circulation.

[5] Shah M, Jorde UP (2019). Percutaneous mitral valve interventions (repair): current indications and future perspectives. Front Cardiovasc Med.

[6] Lazam S, Vanoverschelde JL, Tribouilloy C, Grigioni F, Suri RM, Avierinos JF, de Meester C, Barbieri A, Rusinaru D, Russo A, Pasquet A, Michelena HI, Huebner M, Maalouf J, Clavel MA, Szymanski C, Enriquez-Sarano M (2017) MIDA (Mitral Regurgitation International Database) Investigators. Twenty-year outcome after mitral repair versus replacement for severe degenerative mitral regurgitation: analysis of a large, prospective, multicenter, international registry. Circulation. 135(5):410–422 10.1161/CIRCULATIONAHA.116.023340 27899396

[7] Suri RM, Vanoverschelde JL, Grigioni F, Schaff HV, Tribouilloy C, Avierinos JF, Barbieri A, Pasquet A, Huebner M, Rusinaru D, Russo A, Michelena HI, Enriquez-Sarano M (2013). Association between early surgical intervention vs watchful waiting and outcomes for mitral regurgitation due to flail mitral valve leaflets. JAMA.

[8] Enriquez-Sarano M (2002). Timing of mitral valve surgery. Heart.

[9] Ling H, Enriquez-Sarano M, Seward J (1996). Clinical outcome of mitral regurgitation due to flail leaflets. N Engl J Med.

[10] Del Rio JM, Grecu L, Nicoara A (2019). Right ventricular function in left heart disease. Semin Cardiothorac Vasc Anesth.

[11] Ma JI, Igata S, Strachan M, Nishimura M, Wong DJ, Raisinghani A, DeMaria AN (2019). Predictive factors for progression of mitral regurgitation in asymptomatic patients with mitral valve prolapse. Am J Cardiol.

[12] Mauri L, Garg P, Massaro JM (2010). The EVEREST II Trial: design and rationale for a randomized study of the evalve mitraclip system compared with mitral valve surgery for mitral regurgitation. Am Heart J.

[13] Sorajja P, Mack M, Vemulapalli S, Holmes DR, Stebbins A, Kar S, Lim DS, Thourani V, McCarthy P, Kapadia S, Grayburn P, Pedersen WA, Ailawadi G (2016). Initial experience with commercial transcatheter mitral valve repair in the United States. J Am Coll Cardiol.

[14] Nickenig G, Estevez-Loureiro R, Franzen O, Tamburino C, Vanderheyden M, Lüscher TF, Moat N, Price S, Dall'Ara G, Winter R, Corti R, Grasso C, Snow TM, Jeger R, Blankenberg S, Settergren M, Tiroch K, Balzer J, Petronio AS, Büttner HJ, Ettori F, Sievert H, Fiorino MG, Claeys M, Ussia GP, Baumgartner H, Scandura S, Alamgir F, Keshavarzi F, Colombo A, Maisano F, Ebelt H, Aruta P, Lubos E, Plicht B, Schueler R, Pighi M, Di Mario C (2014) Transcatheter valve treatment sentinel registry investigators of the EURObservational Research Programme of the European Society of Cardiology. Percutaneous mitral valve edge-to-edge repair: in-hospital results and 1-year follow-up of 628 patients of the 2011–2012 Pilot European Sentinel Registry. J Am Coll Cardiol 64(9):875–84 10.1016/j.jacc.2014.06.1166 25169171

[15] Lim DS, Reynolds MR, Feldman T, Kar S, Herrmann HC, Wang A, Whitlow PL, Gray WA, Grayburn P, Mack MJ, Glower DD (2014). Improved functional status and quality of life in prohibitive surgical risk patients with degenerative mitral regurgitation after transcatheter mitral valve repair. J Am Coll Cardiol.

[16] Grayburn PA, Foster E, Sangli C, Weissman NJ, Massaro J, Glower DG, Feldman T, Mauri L (2013). Relationship between the magnitude of reduction in mitral regurgitation severity and left ventricular and left atrial reverse remodeling after MitraClip therapy. Circulation.

[17] Coutinho GF, Antunes MJ (2017). Mitral valve repair for degenerative mitral valve disease: surgical approach, patient selection and long-term outcomes. Heart.

[18] Cameli M, Mandoli GE, Sciaccaluga C, Mondillo S (2019). More than 10 years of speckle tracking echocardiography: still a novel technique or a definite tool for clinical practice?. Echocardiography.

[19] Pastore MC, De Carli G, Mandoli GE, D'Ascenzi F, Focardi M, Contorni F, Mondillo S, Cameli M (2020). The prognostic role of speckle tracking echocardiography in clinical practice: evidence and reference values from the literature. Heart Fail Rev.

[20] Marques-Alves P, Marinho AV, Domingues C, Baptista R, Castro G, Martins R, Gonçalves L (2020). Left atrial mechanics in moderate mitral valve disease: earlier markers of damage. Int J Cardiovasc Imaging.

[21] Kislitsina ON, Thomas JD, Crawford E, Michel E, Kruse J, Liu M, Andrei AC, Cox JL, McCarthy PM (2020). Predictors of left ventricular dysfunction after surgery for degenerative mitral regurgitation. Ann Thorac Surg.

[22] Lancellotti P, Cosyns B, Zacharakis D, Attena E, Van Camp G, Gach O, Radermecker M, Piérard LA (2008). Importance of left ventricular longitudinal function and functional reserve in patients with degenerative mitral regurgitation: assessment by two-dimensional speckle tracking. J Am Soc Echocardiogr.

[23] Cho EJ, Park SJ, Yun HR, Jeong DS, Lee SC, Park SW, Park PW (2016). Predicting left ventricular dysfunction after surgery in patients with chronic mitral regurgitation: assessment of myocardial deformation by 2-dimensional multilayer speckle tracking echocardiography. Korean Circ J.

[24] Witkowski TG, Thomas JD, Debonnaire PJ, Delgado V, Hoke U, Ewe SH, Versteegh MI, Holman ER, Schalij MJ, Bax JJ, Klautz RJ, Marsan NA (2013). Global longitudinal strain predicts left ventricular dysfunction after mitral valve repair. Eur Heart J Cardiovasc Imaging.

[25] Kim HM, Cho GY, Hwang IC, Choi HM, Park JB, Yoon YE, Kim HK (2018). Myocardial strain in prediction of outcomes after surgery for severe mitral regurgitation. JACC Cardiovasc Imaging.

[26] Hiemstra YL, Tomsic A, van Wijngaarden SE, Palmen M, Klautz RJM, Bax JJ, Delgado V, Ajmone MN (2020). Prognostic value of global longitudinal strain and etiology after surgery for primary mitral regurgitation. JACC Cardiovasc Imaging.

[27] Singh V, Kumar S, Bhandari M, Devenraj V, Singh SK (2020). Global longitudinal strain: is it a superior assessment method for left ventricular function in patients with chronic mitral regurgitation undergoing mitral valve replacement?. Indian J Thorac Cardiovasc Surg.

[28] Alashi A, Mentias A, Patel K (2016). Synergistic utility of brain natriuretic peptide and left ventricular global longitudinal strain in asymptomatic patients with significant primary mitral regurgitation and preserved systolic function undergoing mitral valve surgery. Circ Cardiovasc Imaging.

[29] Canessa M, Thamman R, Americo C, Soca G, Dayan V (2020). Global longitudinal strain predicts survival and left ventricular function after mitral valve surgery. A meta-analysis Semin Thorac Cardiovasc Surg.

[30] Mentias A, Naji P, Gillinov AM, Rodriguez LL, Reed G, Mihaljevic T, Suri RM, Sabik JF, Svensson LG, Grimm RA, Griffin BP, Desai MY (2016). Strain echocardiography and functional capacity in asymptomatic primary mitral regurgitation with preserved ejection fraction. J Am Coll Cardiol.

[31] Fukui M, Niikura H, Sorajja P, Hashimoto G, Bae R, Garcia S, Gössl M, Cavalcante JL (2020). Identification of subclinical myocardial dysfunction and association with survival after transcatheter mitral valve repair. J Am Soc Echocardiogr.

[32] Pandis D, Sengupta PP, Castillo JG, Caracciolo G, Fischer GW, Narula J, Anyanwu A, Adams DH (2014). Assessment of longitudinal myocardial mechanics in patients with degenerative mitral valve regurgitation predicts postoperative worsening of left ventricular systolic function. J Am Soc Echocardiogr.

[33] Mascle S, Schnell F, Thebault C, Corbineau H, Laurent M, Hamonic S, Veillard D, Mabo P, Leguerrier A, Donal E (2012). Predictive value of global longitudinal strain in a surgical population of organic mitral regurgitation. J Am Soc Echocardiogr.

[34] Mihaila S, Muraru D, Miglioranza MH, Piasentini E, Aruta P, Cucchini U, Iliceto S, Vinereanu D, Badano LP (2016). Relationship between mitral annulus function and mitral regurgitation severity and left atrial remodelling in patients with primary mitral regurgitation. Eur Heart J Cardiovasc Imaging.

[35] Cameli M, Mandoli GE, Nistor D, Lisi E, Massoni A, Crudele F, Stricagnoli M, Lunghetti S, Mondillo S (2018). Left heart longitudinal deformation analysis in mitral regurgitation. Int J Cardiovasc Imaging.

[36] Cameli M, Lisi M, Righini FM, Massoni A, Natali BM, Focardi M, Tacchini D, Geyer A, Curci V, Di Tommaso C, Lisi G, Maccherini M, Chiavarelli M, Massetti M, Tanganelli P, Mondillo S (2013). Usefulness of atrial deformation analysis to predict left atrial fibrosis and endocardial thickness in patients undergoing mitral valve operations for severe mitral regurgitation secondary to mitral valve prolapse. Am J Cardiol.

[37] Cameli M, Lisi M, Giacomin E, Caputo M, Navarri R, Malandrino A, Ballo P, Agricola E, Mondillo S (2011). Chronic mitral regurgitation: left atrial deformation analysis by two-dimensional speckle tracking echocardiography. Echocardiography.

[38] Vieira MJ, Teixeira R, Gonçalves L et al (2014) Left atrial mechanics: echocardiographic assessment and clinical implications. J Am Soc Echocardiogr 27:463e78 10.1016/j.echo.2014.01.021 24656882

[39] Her AY, Choi EY, Shim CY, Song BW, Lee S, Ha JW, Rim SJ, Hwang KC, Chang BC, Chung N (2012). Prediction of left atrial fibrosis with speckle tracking echocardiography in mitral valve disease: a comparative study with histopathology. Korean Circ J.

[40] Cameli M, Miglioranza MH, Magne J, Mandoli GE, Benfari G, Ancona R (2020). Multicentric atrial strain comparison between two different modalities: MASCOT HIT study. Diagnostics (Basel).

[41] Voigt JU, Mălăescu GG, Haugaa K, Badano L (2020). How to do LA strain. Eur Heart J Cardiovasc Imaging.

[42] Yang LT, Shih JY, Liu YW et al (2013) Effects of left atrial strain on functional capacity in chronic severe mitral regurgitation. Int J Cardiol 168:e151e3 10.1016/j.ijcard.2013.08.070 24035177

[43] Mandoli GE, Pastore MC, Benfari G, Bisleri G, Maccherini M, Lisi G, Cameli P, Lisi M, Dokollari A, Carrucola C, Vigna M, Montesi G, Valente S, Mondillo S, Cameli M (2020). Left atrial strain as a pre-operative prognostic marker for patients with severe mitral regurgitation. Int J Cardiol.

[44] Cameli M, Pastore MC, Righini FM, Mandoli GE, D'Ascenzi F, Lisi M, Nistor D, Sparla S, Curci V, Di Tommaso C, Marino F, Stricagnoli M, Mondillo S (2019). Prognostic value of left atrial strain in patients with moderate asymptomatic mitral regurgitation. Int J Cardiovasc Imaging.

[45] Toprak C, Kahveci G, Kilicgedik A, Pala S, Kirma C, Tabakci MM, Inanir M, Esen AM (2016). Left atrial remodeling in patients undergoing percutaneous mitral valve repair with the MitraClip system: an advanced echocardiography study. Echocardiography.

[46] Kamijima R, Suzuki K, Izumo M, Kuwata S, Mizukoshi K, Takai M, Kou S, Hayashi A, Kida K, Harada T, Akashi YJ (2017). Predictors of exercise-induced pulmonary hypertension in patients with asymptomatic degenerative mitral regurgitation: mechanistic insights from 2D speckle-tracking echocardiography. Sci Rep.

[47] Sugimoto T, Bandera F, Generati G, Alfonzetti E, Barletta M, Losito M, Labate V, Rovida M, Caracciolo M, Pappone C, Ciconte G, Guazzi M (2020). Left atrial dynamics during exercise in mitral regurgitation of primary and secondary origin: pathophysiological insights by exercise echocardiography combined with gas exchange analysis. JACC Cardiovasc Imaging.

[48] Debonnaire P, Leong DP, Witkowski TG, Al Amri I, Joyce E, Katsanos S, Schalij MJ, Bax JJ, Delgado V, Marsan NA (2013). Left atrial function by two-dimensional speckle-tracking echocardiography in patients with severe organic mitral regurgitation: association with guidelines-based surgical indication and postoperative (long-term) survival. J Am Soc Echocardiogr.

[49] Ring L, Abu-Omar Y, Kaye N, Rana BS, Watson W, Dutka DP, Vassiliou VS (2018). Left atrial function is associated with earlier need for cardiac surgery in moderate to severe mitral regurgitation: usefulness in targeting for early surgery. J Am Soc Echocardiogr.

[50] Dzeshka MS, Lip GY, Snezhitskiy V, Shantsila E (2015). Cardiac fibrosis in patients with atrial fibrillation: mechanisms and clinical implications. J Am Coll Cardiol.

[51] Cameli M, Mandoli GE, Loiacono F, Sparla S, Iardino E, Mondillo S (2016) Left atrial strain: a useful index in atrial fibrillation. Int J Cardiol; 220:208–13. 10.1016/j.ijcard.2016.06.197 27389443

[52] Cameli M, Lisi M, Righini FM, Focardi M, Alfieri O, Mondillo S (2012). Left atrial speckle tracking analysis in patients with mitral insufficiency and history of paroxysmal atrial fibrillation. Int J Cardiovasc Imaging.

[53] Candan O, Ozdemir N, Aung SM et al (2013) Left atrial longitudinal strain parameters predict postoperative persistent atrial fibrillation following mitral valve surgery: a speckle tracking echocardiography study. Echocardiography 30:1061e8 10.1111/echo.12222 23600893

[54] Candan O, Ozdemir N, Aung SM et al (2014) Atrial longitudinal strain parameters predict left atrial reverse remodeling after mitral valve surgery: a speckle tracking echocardiography study. Int J Cardiovasc Imaging 30:1049e56 10.1007/s10554-014-0433-9 24781032

[55] Yang LT, Tsai WC, Luo CY, Li YH, Tsai LM (2017). Role of left atrial reservoir strain rate in left atrial remodeling in severe mitral regurgitation. J Med Ultrasound.

[56] Badano LP, Kolias TJ, Muraru D, Abraham TP, Aurigemma G, Edvardsen T, D'Hooge J, Donal E, Fraser AG, Marwick T, Mertens L, Popescu BA, Sengupta PP, Lancellotti P, Thomas JD, Voigt JU (2018) Industry representatives; Reviewers: This document was reviewed by members of the 2016–2018 EACVI Scientific Documents Committee. Standardization of left atrial, right ventricular, and right atrial deformation imaging using two-dimensional speckle tracking echocardiography: a consensus document of the EACVI/ASE/Industry Task Force to standardize deformation imaging. Eur Heart J Cardiovasc Imaging. 19(6):591–600. 10.1093/ehjci/jey042. Erratum in: Eur Heart J Cardiovasc Imaging. 2018 Jul 1;19(7):830–833 10.1093/ehjci/jey042 29596561

[57] Bakkestrøm R, Banke A, Christensen NL, Pecini R, Irmukhamedov A, Andersen M, Borlaug BA, Møller JE (2018). Hemodynamic characteristics in significant symptomatic and asymptomatic primary mitral valve regurgitation at rest and during exercise. Circ Cardiovasc Imaging.

[58] Sabe MA, Sabe SA, Kusunose K, Flamm SD, Griffin BP, Kwon DH (2016). Predictors and prognostic significance of right ventricular ejection fraction in patients with ischemic cardiomyopathy. Circulation.

[59] Mandoli GE, Cameli M, Novo G, Agricola E, Righini FM, Santoro C, D'Ascenzi F, Ancona F, Sorrentino R, D'Andrea A, Galderisi M, Mondillo S (2019) Working Group of Echocardiography of the Italian Society of Cardiology. Right ventricular function after cardiac surgery: the diagnostic and prognostic role of echocardiography. Heart Fail Rev 24(5):625–635 10.1007/s10741-019-09785-2 30982175

[60] Chrustowicz A, Gackowski A, El-Massri N, Sadowski J, Piwowarska W (2010). Preoperative right ventricular function in patients with organic mitral regurgitation. Echocardiography.

[61] Chrustowicz A, Simonis G, Matschke K, Strasser RH, Gackowski A (2009). Right ventricular dilatation predicts survival after mitral valve repair in patients with impaired left ventricular systolic function. Eur J Echocardiogr.

[62] Mordi I, Al-Attar N, Tzemos N (2014). Preoperative assessment of left ventricular diastolic function and right ventricular systolic function have independent and incremental prognostic value in prediction of early postoperative mortality in redo valve surgery. Echocardiography.

[63] Pastore MC, Mandoli GE, Aboumarie HS, Santoro C, Bandera F, D'Andrea A, Benfari G, Esposito R, Evola V, Sorrentino R, Cameli P, Valente S, Mondillo S, Galderisi M, Cameli M (2020) Working Group of Echocardiography of the Italian Society of Cardiology. Basic and advanced echocardiography in advanced heart failure: an overview. Heart Fail Rev 25(6):937–948. 10.1007/s10741-019-09865-3. 10.1007/s10741-019-09865-3 31617033

[64] Bellavia D, Iacovoni A, Scardulla C, Moja L, Pilato M, Kushwaha SS, Senni M, Clemenza F, Agnese V, Falletta C, Romano G, Maalouf J, Dandel M (2017). Prediction of right ventricular failure after ventricular assist device implant: systematic review and meta-analysis of observational studies. Eur J Heart Fail.

[65] Nabeshima Y, Seo Y, Takeuchi M (2020). A review of current trends in three-dimensional analysis of left ventricular myocardial strain. Cardiovasc Ultrasound.

[66] Casas-Rojo E, Fernández-Golfin C, Moya-Mur JL, González-Gómez A, García-Martín A, Morán-Fernández L, Rodríguez-Muñoz D, Jiménez-Nacher JJ, Martí Sánchez D, Zamorano Gómez JL (2016). Area strain from 3D speckle-tracking echocardiography as an independent predictor of early symptoms or ventricular dysfunction in asymptomatic severe mitral regurgitation with preserved ejection fraction. Int J Cardiovasc Imaging.

[67] Vitarelli A, Mangieri E, Capotosto L, Tanzilli G, D'Angeli I, Viceconte N, Placanica A, Placanica G, Cocco N, Ashurov R, Al-Kindy S (2015). Assessment of biventricular function by three-dimensional speckle-tracking echocardiography in secondary mitral regurgitation after repair with the MitraClip system. J Am Soc Echocardiogr.

[68] Scandura S, Dipasqua F, Gargiulo G, Capodanno D, Caggegi A, Grasso C, Mangiafico S, Pistritto AM, Immè S, Chiarandà M, Ministeri M, Ronsivalle G, Cannata S, Arcidiacono AA, Capranzano P, Tamburino C (2016). Early results of MitraClip system implantation by real-time three-dimensional speckle-tracking left ventricle analysis. J Cardiovasc Med (Hagerstown).

